# Preoperative weight loss before total hip arthroplasty negatively impacts postoperative outcomes

**DOI:** 10.1186/s42836-024-00237-3

**Published:** 2024-04-02

**Authors:** Jessica Schmerler, Nauman Hussain, Shyam J. Kurian, Harpal S. Khanuja, Julius K. Oni, Vishal Hegde

**Affiliations:** Department of Orthopaedic Surgery, The Johns Hopkins University School of Medicine, 601 N Caroline St, Baltimore, MD 21287 USA

**Keywords:** Total hip arthroplasty, Complications, BMI, Weight loss, Obesity

## Abstract

**Background:**

Obesity adversely impacts outcomes of total hip arthroplasty (THA), leading surgeons to impose body mass index cutoffs for patient eligibility and encourage preoperative weight loss. This study aimed to determine if preoperative weight loss impacts outcomes of THA in the general patient population and if it mitigates poor outcomes in obese patients.

**Methods:**

Patients who underwent THA from 2013–2020 were identified in the National Surgical Quality Improvement Program (NSQIP) database. Patients were stratified by weight loss of >10% of body weight over the preceding 6 months. We used multivariable linear and logistic regression models, adjusted for age, sex, race/ethnicity, and comorbidities, to examine the effect of significant preoperative weight loss on 30-day outcomes after THA in the general and obese patient populations.

**Results:**

In the overall population, patients who lost significant weight preoperatively had significantly increased length of stay, were more likely to have a non-home discharge, return to the operating room, or be readmitted, and were more likely to experience numerous medical complications. In the obese population, patients who lost significant weight preoperatively had significantly increased length of stay and were more likely to require a transfusion or experience any medical complication.

**Discussion:**

Rapid significant preoperative weight loss is not associated with improved postoperative outcomes after THA in the obese population and is associated with worse outcomes in the general population. Arthroplasty surgeons should balance these risks with the risks of obesity when advising patients about preoperative weight loss prior to THA.

**Level of evidence:**

III.

## Introduction

Total hip arthroplasty (THA) remains an effective procedure for the management of chronic hip pain and limited mobility due to arthritis, with over 370,000 procedures performed in 2014 [1]. This number is projected to increase by 71.2% to 635,000 procedures annually by 2030 [1]. According to recent studies, the prevalence of obesity in patients undergoing THA has risen to 47.1% in the United States [2]. Obesity is associated with several surgical risk factors after total joint arthroplasty (TJA), including increased rates of pulmonary embolism, acute kidney injury, cardiac arrest, reintubation, superficial infection, and reoperation [3–5]. In addition to higher risks of postoperative complications, obese patients have a higher prevalence of comorbidities such as hypertension, diabetes, and cardiovascular disease [6]. Thus, in an effort to reduce complications after THA, many orthopaedic surgeons have begun instituting body mass index (BMI) cutoffs for eligibility for THA, for example, 35 kg/m^2^ (class II obesity) or 40 kg/m^2^(class III obesity) [7, 8].

Malnutrition is common in obesity, and weight loss may exacerbate the effects of malnutrition, such as impaired wound healing and increased infection risk postoperatively [9]. Given the potential for a compounding effect, the question arises of whether significant preoperative weight loss confers benefits or exerts harmful effects in the general and obese populations, and if these effects differ. Numerous studies have investigated the impact of bariatric surgery prior to TJA on postoperative outcomes and findings have been inconsistent, with some showing reduced risk for adverse outcomes and others showing no significant differences [10, 11]. For non-surgical weight loss, one study demonstrated that individuals with a BMI > 40 kg/m^2^ who lost weight to a BMI < 40 kg/m^2^were at greater risk for readmission and complications than individuals below that BMI cutoff [12]. However, this study did not have sufficient data to compare individuals who lost weight to those who remained at a BMI > 40 kg/m^2^, nor did it investigate weight loss across the BMI spectrum. Another study demonstrated that patients with a BMI of >30 kg/m^2 ^who lost ≥5% of body weight in the year before THA had an increased risk of deep surgical site infection compared to other obese patients [13], but this study examined only two outcomes and did not control for comorbidities or patient characteristics other than BMI.

No studies, to our knowledge, have investigated the association between significant weight loss prior to THA and postoperative outcomes in non-obese patients. Additionally, we seek to expand the existing literature in obese patients by investigating if rapid, significant preoperative weight loss impacts a broad array of outcomes after THA, controlling for comorbidities and patient characteristics. We defined significant weight loss as >10% of body weight, as this has been associated with risk for malnutrition [14]. Given the inherent risks of weight loss and malnutrition, we hypothesized that significant preoperative weight loss would adversely impact 30-day outcomes of THA. Understanding this impact will help arthroplasty surgeons balance the risks of preoperative weight loss prior to THA with the risks of performing THA in obese patients.

## Methods

This study was exempt from IRB approval.

### Data source and study population

The American College of Surgeons National Surgical Quality Improvement Program (NSQIP) database was utilized to identify patients of 18 years or older who underwent THA from 1 January 2013 to 31 December 2020 using the Current Procedural Terminology code 27130. Patients with cancer diagnoses were excluded. Patients were stratified by an NSQIP variable for malnutrition, defined as weight loss of >10% of body weight over the preceding 6 months. A subgroup analysis was then conducted, isolating patients who were obese either at the time of operation or within 6 months prior. Minimum pre-weight loss BMI was calculated for patients who had lost >10% of body weight by determining their minimum weight prior to weight loss (weight at the time of operation divided by 0.9) and then calculating BMI as: (weight in pounds)/(height in inches)^2^ × 703. Patient weight and height utilized were specific values drawn from patient charts collected in the NSQIP database, and only patients for whom preoperative weight and height were available were included.

### Variables of interest

#### Outcomes of interest

The primary outcomes were differences in operative characteristics (length of stay [LOS], operative time, discharge destination, return to OR, and readmission) and likelihood of 30-day postoperative complications after THA between patients who underwent significant preoperative weight loss and patients who did not. LOS was defined as time from operation to discharge. Complications investigated included individual medical complications (surgical site infections, wound dehiscence, pneumonia, reintubation, pulmonary embolism, failure to wean, renal insufficiency, renal failure, urinary tract infection, stroke, cardiac arrest, myocardial infarction, bleeding complication, deep venous thrombosis, sepsis, and septic shock) and a combined variable for any medical complication.

#### Covariates

Included as covariates were age, sex, race/ethnicity, ASA classification, inpatient vs. outpatient procedure, diabetes, smoking status, congestive heart failure (CHF), hypertension medication usage, functional status, and weight. Additional relevant covariates were also included for specific outcomes, namely, preoperative wound infections for postoperative infectious complications, preoperative immunosuppressive use for postoperative pneumonia, urinary tract infections, or septic complications, preoperative renal failure or dialysis for renal complications, and preoperative bleeding disorders for postoperative bleeding complications or deep venous thrombosis.

### Statistical analysis

Differences in discharge destination, return to OR, readmission, and postoperative complications were analyzed using univariate chi-square analysis, and differences in LOS and operative time using two-tailed *t*-tests. Multivariable logistic and linear regression models (depending on whether the outcome was categorical or continuous, respectively) controlling for the above-listed covariates were then constructed to examine the effect of significant preoperative weight loss on each outcome. Data were analyzed using Stata Statistical Software: Release 17; 2021 (StataCorp; College Station, TX, USA). A *P* value of <0.05 was considered statistically significant.

## Results

### Patient characteristics

A total of 265,496 patients underwent THA from 2013 to 2020. Of these, 567 (0.2%) lost significant weight preoperatively. Table 1 presents the characteristics of patients undergoing THA, stratified by preoperative weight loss. Women accounted for 54.9% of the patient population, and patients were on average 65.4 ± 11.4 years old. Age, sex, weight and BMI, preoperative albumin and malnutrition (albumin < 3.5 g/dL), ASA class, functional status, diabetes status, smoking status, history of CHF, and hypertensive medication usage all varied significantly by preoperative weight loss (*P* < 0.05 all).

**Table 1:** Demographic information on patients undergoing THA, stratified by preoperative weight loss of >10% of body weight in 6 months prior to surgery

**Characteristic**	**Total Sample**	**No Weight Loss**	**Weight Loss**	***P*-value**
*N* (%)	265,496	264,929 (99.8%)	567 (0.2%)	
Age (SD)	65.4 (11.4)	65.4 (11.4)	69.1 (12.0)	<0.001
Sex				0.001
Women	145,714 (54.9%)	145,363 (54.8%)	351 (61.8%)	
Men	119,943 (45.1%)	119,726 (45.2%)	217 (38.2%)	
Race/Ethnicity				0.98
Non-Hispanic White	186,136 (70.1%)	185,735 (70.1%)	401 (70.6%)	
Non-Hispanic Black	20,847 (7.9%)	20,807 (7.9%)	40 (7.0%)	
Hispanic	8,438 (3.2%)	8,418 (3.2%)	20 (3.5%)	
Non-Hispanic Asian	3.993 (1.5%)	3.985 (1.5%)	8 (1.4%)	
Non-Hispanic Other	1,684 (0.6%)	1,680 (0.6%)	4 (0.7%)	
Race/ Ethnicity Unknown	44,559 (16.8%)	44,464 (16.8%)	95 (16.7%)	
Weight (lbs) (SD)	189.0 (45.9)	189.0 (45.9)	156.4 (50.5)	<0.001
BMI (kg/m^2^) (SD)	30.2 (6.3)	30.2 (6.3)	25.5 (7.1)	<0.001
Preoperative Albumin Level (SD)	4.14 (0.42)	4.14 (0.42)	3.72 (0.60)	<0.001
Malnutrition (Albumin < 3.5 g/dL)				<0.001
Yes	7,158 (5.1%)	7,054 (5.1%)	104 (30.8%)	
No	132,126 (94.9%)	131,892 (94.9%)	234 (69.2%)	
Setting				0.12
Inpatient	247,725 (93.2%)	247,186 (93.2%)	539 (94.9%)	
Outpatient	17,932 (6.8%)	17,903 (6.8%)	29 (5.1%)	
ASA Class				<0.001
1	9,533 (3.6%)	9,527 (3.6%)	6 (1.1%)	
2	137,777 (51.9%)	137,605 (51.9%)	172 (30.3%)	
3	112,428 (42.3%)	112,092 (42.3%)	336 (59.2%)	
4	5,606 (2.1%)	5,553 (2.1%)	53 (9.3%)	
5	21 (0.01%)	21 (0.01%)	0 (0.0%)	
Unknown	292 (0.1%)	291 (0.1%)	1 (0.2%)	
Functional Status				<0.001
Independent	259,477 (97.7%)	258,988 (97.7%)	489 (86.1%)	
Partially Dependent	4,799 (1.8%)	4,730 (1.8%)	69 (12.2%)	
Totally Dependent	286 (0.1%)	277 (0.1%)	9 (1.6%)	
Unknown	1,095 (0.4%)	1,094 (0.4%)	1 (0.2%)	
Diabetes				0.03
Yes	32,483 (12.2%)	32,397 (12.2%)	86 (15.1%)	
No	233,174 (87.8%)	232,692 (87.8%)	482 (84.9%)	
Smoking Status				<0.001
Smoker	33,093 (12.5%)	32,967 (12.4%)	126 (22.2%)	
Non-Smoker	232,564 (87.5%)	232,122 (87.6%)	442 (77.8%)	
History of CHF				<0.001
Yes	1,058 (0.4%)	1,045 (0.4%)	13 (2.4%)	
No	264,599 (99.6%)	264,044 (99.6%)	555 (97.7%)	
On Medication for Hypertension				0.002
Yes	146,558 (55.2%)	146,208 (55.2%)	350 (61.6%)	
No	119,099 (44.8%)	118,881 (44.8%)	218 (38.4%)	
Renal Failure				<0.001
Yes	173 (0.1%)	170 (0.1%)	3 (0.5%)	
No	265,484 (99.9%)	264,919 (99.9%)	565 (99.5%)	
Dialysis				<0.001
Yes	676 (0.3%)	668 (0.3%)	8 (1.4%)	
No	264,981 (99.7%)	264,421 (99.7%)	560 (98.6%)	
Preoperative Wound Infection				<0.001
Yes	849 (0.3%)	827 (0.3%)	22 (3.9%)	
No	264,808 (99.7%)	264,262 (99.7%)	546 (96.1%)	
On Immuno-suppressive Therapy				<0.001
Yes	9,865 (3.7%)	9,808 (3.7%)	57 (10.0%)	
No	255,792 (96.3%)	255,281 (96.3%)	511 (90.0%)	
Bleeding Disorder				<0.001
Yes	5,798 (2.2%)	5,766 (2.2%)	32 (5.6%)	
No	259,859 (97.8%)	259,323 (97.8%)	536 (94.4%)	

Of the 265,496 patients who underwent THA over 2013–2020, a subset of 54,491 patients were obese within 6 months preoperatively. Of these, 103 (0.2%) lost significant weight preoperatively. Table 2 presents the characteristics of obese patients undergoing THA, stratified by preoperative weight loss. Women accounted for 54.9% of the patient population, and patients were on average 62.3 ± 10.2 years old. Weight and BMI, preoperative albumin and malnutrition, functional status, and history of CHF varied significantly by preoperative weight loss (*P* < 0.05 for all).

**Table 2:** Demographic information on patients undergoing THA with a BMI > 35, stratified by preoperative weight loss of >10% of body weight in 6 months prior to surgery

**Characteristic**	**Total Sample**	**No Weight Loss**	**Weight Loss**	***P*-value**
*N* (%)	54,491	54,388 (99.8%)	103 (0.2%)	
Age (SD)	62.3 (10.2)	62.3 (10.2)	63.9 (10.1)	0.11
Sex				0.63
Women	29,920 (54.9%)	29,861 (54.9%)	59 (57.3%)	
Men	24,571 (45.1%)	24,527 (45.1%)	44 (42.7%)	
Race/Ethnicity				0.14
Non-Hispanic White	38,133 (70.0%)	38,055 (70.0%)	78 (75.7%)	
Non-Hispanic Black	5,879 (10.8%)	5,874 (10.8%)	5 (4.9%)	
Hispanic	1,850 (3.4%)	1,847 (3.4%)	3 (2.9%)	
Non-Hispanic Asian	269 (0.5%)	267 (0.5%)	2 (1.9%)	
Non-Hispanic Other	462 (0.9%)	461 (0.9%)	1 (1.0%)	
Race/ Ethnicity Unknown	7,898 (14.5%)	7,884 (14.5%)	14 (13.6%)	
Weight (lbs) (SD)	245.4 (38.9)	245.4 (38.9)	234.0 (44.8)	0.003
BMI (kg/m^2^) (SD)	39.5 (4.3)	39.5 (4.3)	37.5 (5.0)	<0.001
Preoperative Albumin Level (SD)	4.11 (0.41)	4.11 (0.41)	3.90 (0.53)	<0.001
Malnutrition (Albumin <3.5g/dL)				<0.001
Yes	1,421 (4.8%)	1,412 (4.8%)	9 (14.5%)	
No	28,276 (95.2%)	28,223 (95.2%)	53 (85.5%)	
Setting				0.59
Inpatient	51,151 (93.9%)	51,053 (93.9%)	98 (95.1%)	
Outpatient	3,340 (6.1%)	3,335 (6.1%)	5 (4.9%)	
ASA Class				0.07
1	367 (0.7%)	367 (0.7%)	0 (0.0%)	
2	18,707 (34.3%)	18,676 (34.3%)	31 (30.1%)	
3	33,826 (62.1%)	33,762 (62.1%)	64 (62.1%)	
4	1,535 (2.8%)	1,527 (2.8%)	8 (7.8%)	
5	21 (0.01%)	21 (0.01%)	0 (0.0%)	
Unknown	50 (0.1%)	50 (0.1%)	0 (0.0%)	
Functional Status				0.01
Independent	53,125 (97.5%)	53,029 (97.5%)	96 (93.2%)	
Partially Dependent	1,074 (2.0%)	1,067 (2.0%)	7 (6.8%)	
Totally Dependent	31 (0.1%)	31 (0.1%)	0 (0.0%)	
Unknown	261 (0.5%)	261 (0.4%)	0 (0.0%)	
Diabetes				0.25
Yes	11,760 (21.6%)	11,733 (21.6%)	27 (26.2%)	
No	42,731 (78.4%)	42,655 (78.4%)	76 (73.8%)	
Smoking Status				0.40
Smoker	6,465 (11.9%)	6,450 (11.9%)	15 (14.6%)	
Non-Smoker	48,026 (88.1%)	47,938 (88.1%)	88 (85.4%)	
History of CHF				0.03
Yes	266 (0.5%)	264 (0.5%)	2 (1.9%)	
No	54,225 (99.5%)	54,124 (99.5%)	101 (98.1%)	
On Medication for Hypertension				0.16
Yes	37,391 (68.6%)	37,327 (68.6%)	64 (62.1%)	
No	17,100 (31.4%)	17,061 (31.4%)	39 (37.9%)	
Renal Failure				<0.001
Yes	28 (0.1%)	26 (0.1%)	2 (1.9%)	
No	54,463 (99.9%)	54,362 (99.9%)	101 (98.1%)	
Dialysis				0.11
Yes	124 (0.2%)	123 (0.2%)	1 (1.0%)	
No	54,367 (99.8%)	54,265 (99.8%)	102 (99.0%)	
Preoperative Wound Infection				0.54
Yes	194 (0.4%)	194 (0.4%)	0 (0.0%)	
No	54,297 (99.6%)	54,194 (99.6%)	103 (100.0%)	
On Immuno-suppressive Therapy				0.71
Yes	1,954 (3.6%)	1,951 (3.6%)	3 (2.9%)	
No	52,537 (96.4%)	52,437 (96.4%)	100 (97.1%)	
Bleeding Disorder				0.65
Yes	1,226 (2.2%)	1,223 (2.2%)	3 (2.9%)	
No	53,265 (97.8%)	53,165 (97.8%)	100 (97.1%)	

### Total patient population

Table 3 presents the full results of univariate modeling for the total patient population, and the full results of multivariable modeling can be found in Table 4.

**Table 3:** Univariate analysis of postoperative outcomes stratified by preoperative weight loss in the general population

**Outcome**	**Total Sample**	**No Weight Loss**	**Weight Loss**	***P*-value**
Length of Stay (Days from Operation to Discharge) (Range)	2.24 (0–116)	2.23 (0–116)	3.62 (0–67)	<0.001
Operative Time (Range)	91.4 (0–1387)	91.4 (0–1387)	92.0 (3–300)	0.73
Discharge Destination				<0.001
Home	222,683 (83.8%)	222,339 (83.9%)	344 (60.6%)	
Non-Home	41,602 (15.7%)	41,395 (15.6%)	207 (36.4%)	
Unknown	1,372 (0.5%)	1,355 (0.5%)	17 (3.0%)	
Return to OR				0.001
Yes	5,052 (1.9%)	5,030 (1.9%)	22 (3.9%)	
No	260,605 (98.1%)	260,059 (98.1%)	546 (96.1%)	
Still in Hospital after 30 days				0.002
Yes	145 (0.1%)	143 (0.1%)	2 (0.3%)	
No	265,512 (99.9%)	264,946 (99.9%)	566 (99.7%)	
Readmission				<0.001
Yes	8,990 (3.4%)	8,941 (3.4%)	49 (8.6%)	
No	256,667 (96.6%)	256,148 (96.6%)	519 (91.4%)	
Superficial SSI				0.65
Yes	1,827 (0.7%)	1,824 (0.7%)	3 (0.5%)	
No	263,830 (99.3%)	263,265 (99.3%)	565 (99.5%)	
Deep SSI				0.10
Yes	559 (0.2%)	556 (0.2%)	3 (0.5%)	
No	265,098 (99.8%)	264,533 (99.8%)	565 (99.5%)	
Organ Space SSI				0.09
Yes	817 (0.3%)	813 (0.3%)	4 (0.7%)	
No	264,840 (99.7%)	264,276 (99.7%)	564 (99.3%)	
Wound Dehiscence				0.15
Yes	355 (0.1%)	353 (0.1%)	2 (0.3%)	
No	265,302 (99.9%)	264,736 (99.9%)	566 (99.7%)	
Pneumonia				<0.001
Yes	899 (0.3%)	881 (0.3%)	18 (3.2%)	
No	264,758 (99.7%)	264,208 (99.7%)	550 (96.8%)	
Reintubation				<0.001
Yes	396 (0.1%)	388 (0.1%)	8 (1.4%)	
No	265,261 (99.9%)	264,701 (99.9%)	560 (98.6%)	
Pulmonary Embolism				0.66
Yes	688 (0.3%)	686 (0.3%)	2 (0.3%)	
No	264,969 (99.7%)	264,403 (99.7%)	566 (99.7%)	
Failure to Wean				<0.001
Yes	159 (0.1%)	154 (0.1%)	5 (0.9%)	
No	265,498 (99.9%)	264,935 (99.9%)	563 (99.1%)	
Progressive Renal Insufficiency				<0.001
Yes	235 (0.1%)	232 (0.1%)	3 (0.5%)	
No	265,422 (99.9%)	264,857 (99.9%)	565 (99.5%)	
Acute Renal Failure				0.18
Yes	132 (0.1%)	131 (0.1%)	1 (0.2%)	
No	265,525 (99.9%)	264,958 (99.9%)	567 (99.8%)	
Urinary Tract Infection				0.001
Yes	2,260 (0.9%)	2,248 (0.9%)	12 (2.1%)	
No	263,397 (99.1%)	262,841 (99.1%)	556 (97.9%)	
CVA/Stroke				0.52
Yes	247 (0.1%)	246 (0.1%)	1 (0.2%)	
No	265,410 (99.9%)	264,843 (99.9%)	567 (99.8%)	
Cardiac Arrest				<0.001
Yes	228 (0.1%)	225 (0.1%)	3 (0.5%)	
No	265,429 (99.9%)	264,864 (99.9%)	565 (99.5%)	
Myocardial Infarction				0.002
Yes	629 (0.2%)	624 (0.2%)	5 (0.9%)	
No	265,028 (99.8%)	264,465 (99.8%)	563 (99.1%)	
Bleeding Transfusion				<0.001
Yes	13,484 (5.1%)	13,398 (5.1%)	86 (15.1%)	
No	252,173 (94.9%)	251,691 (94.9%)	482 (84.9%)	
Deep Venous Thrombosis				0.39
Yes	862 (0.3%)	859 (0.3%)	3 (0.5%)	
No	264,795 (99.7%)	264,230 (99.7%)	565 (99.5%)	
Sepsis				<0.001
Yes	622 (0.2%)	616 (0.2%)	6 (1.1%)	
No	265,035 (99.8%)	264,473 (99.8%)	562 (98.9%)	
Septic Shock				<0.001
Yes	169 (0.1%)	163 (0.1%)	6 (1.1%)	
No	265,488 (99.9%)	264,926 (99.9%)	562 (98.9%)	
Any Medical Complication				<0.001
Yes	21,311 (8.0%)	21,188 (8.0%)	123 (21.7%)	
No	244,346 (92.0%)	243,901 (92.0%)	445 (78.3%)

**Table 4:** Multivariable analysis of the impact of preoperative weight loss on postoperative outcomes in the general population

**Outcome**	**Odds Ratio/Coefficient**	**95% CI**	***P*-value**
Length of Stay (Days from Operation to Discharge)	β = 0.90	0.73-1.06	**<0.001**
Non-Home Discharge	OR = 2.04	1.67-2.49	**<0.001**
Return to OR	OR = 1.78	1.16-2.74	**0.01**
Still in Hospital after 30 days	OR = 1.68	0.39-7.31	0.49
Readmission	OR = 1.89	1.39-2.55	**<0.001**
Pneumonia	OR = 3.19	1.94-5.26	**<0.001**
Reintubation	OR = 3.46	1.66-7.21	**0.001**
Failure to Wean	OR = 4.79	1.87-12.29	**0.001**
Progressive Renal Insufficiency	OR = 3.34	1.03-10.89	**0.045**
Urinary Tract Infection	OR = 1.77	0.99-3.16	0.05
Cardiac Arrest	OR = 2.00	0.62-6.47	0.25
Myocardial Infarction	OR = 1.78	0.72-4.37	0.21
Bleeding Transfusion	OR = 1.70	1.33-2.17	**<0.001**
Sepsis	OR = 2.87	1.26-6.57	**0.01**
Septic Shock	OR = 7.40	3.13-17.50	**<0.001**
Any Medical Complication	OR = 1.92	1.56-2.37	**<0.001**

Bold values indicate *P* < 0.05

Patients who lost significant weight preoperatively had significantly increased LOS (β 0.90, 95% CI 0.73–1.06, *P* < 0.001), and were more likely to have a non-home discharge (OR 2.04, 95% CI 1.67–2.49, *P* < 0.001), return to the operating room (OR 1.78, 95% CI 1.16–2.74, *P* = 0.01), and be readmitted (OR 1.89, 95% CI 1.39–2.55, *P* < 0.001). In terms of medical complications, patients who lost significant weight were more likely to experience pneumonia (OR 3.19, 95% CI 1.94–5.26, *P* < 0.001), be reintubated (OR 3.46, 95% CI 1.66–7.21, *P* = 0.001), fail to wean (OR 4.79, 95% CI 1.87–12.29, *P* = 0.001), have progressive renal insufficiency (OR 3.34, 95% CI 1.03–10.89, *P* = 0.045), require a transfusion for bleeding (OR 1.70, 95% CI 1.33–2.17, *P* < 0.001), experience sepsis (OR 2.87, 95% CI 1.26–6.57, *P* = 0.01), experience septic shock (OR = 7.40, 95% CI 3.13–17.50, *P* < 0.001), and experience any medical complication (OR = 1.92, 95% CI 1.56–2.37, *P* < 0.001).

### Obese patient population

Table 5 presents the full results of univariate modeling for patients who were obese preoperatively, and the full results of multivariable modeling can be found in Table 6.

**Table 5:** Univariate analysis of postoperative outcomes in patients with BMI > 35 stratified by preoperative weight loss

**Outcome**	**Total Sample**	**No Weight Loss**	**Weight Loss**	***P*-value**
Length of Stay (Days from Operation to Discharge) (Range)	2.31 (0–116)	2.31 (0–116)	2.83 (0–12)	0.01
Operative Time (Range)	98.6 (0–948)	98.6 (0–948)	101.3 (31–272)	0.49
Discharge Destination				0.22
Home	45,001 (82.6%)	44,922 (82.6%)	79 (76.7%)	
Non-Home	9,273 (17.0%)	9,250 (17.0%)	23 (22.3%)	
Unknown	217 (0.4%)	216 (0.4%)	1 (1.0%)	
Return to OR				0.07
Yes	1,559 (2.9%)	1,553 (2.9%)	6 (5.8%)	
No	52,932 (97.1%)	52,835 (97.1%)	97 (94.2%)	
Still in Hospital after 30 days				0.82
Yes	28 (0.1%)	28 (0.1%)	0 (0.0%)	
No	54,463 (99.9%)	54,360 (99.9%)	103 (100.0%)	
Readmission				0.09
Yes	2,368 (4.3%)	2,360 (4.3%)	8 (7.8%)	
No	52,123 (95.7%)	52,028 (95.7%)	95 (92.2%)	
Superficial SSI				0.24
Yes	707 (1.3%)	707 (1.3%)	0 (0.0%)	
No	53,784 (98.7%)	53,681 (98.7%)	103 (100.0%)	
Deep SSI				0.43
Yes	247 (0.5%)	246 (0.5%)	1 (1.0%)	
No	54,244 (99.5%)	54,142 (99.5%)	102 (99.0%)	
Organ Space SSI				0.70
Yes	361 (0.7%)	360 (0.7%)	1 (1.0%)	
No	54,130 (99.3%)	54,028 (99.3%)	102 (99.0%)	
Wound Dehiscence				0.18
Yes	151 (0.3%)	150 (0.3%)	1 (1.0%)	
No	54,340 (99.7%)	54,238 (99.7%)	102 (99.0%)	
Pneumonia				0.17
Yes	145 (0.3%)	144 (0.3%)	1 (1.0%)	
No	54,346 (99.7%)	54,244 (99.7%)	102 (99.0%)	
Reintubation				0.68
Yes	92 (0.2%)	92 (0.2%)	0 (0.0%)	
No	54,399 (99.8%)	54,296 (99.8%)	103 (100.0%)	
Pulmonary Embolism				0.57
Yes	170 (0.3%)	170 (0.3%)	0 (0.0%)	
No	54,321 (99.7%)	54,218 (99.7%)	103 (100.0%)	
Failure to Wean				0.78
Yes	40 (0.1%)	40 (0.1%)	0 (0.0%)	
No	54,451 (99.9%)	54,348 (99.9%)	103 (100.0%)	
Progressive Renal Insufficiency				0.70
Yes	78 (0.1%)	78 (0.1%)	0 (0.0%)	
No	54,413 (99.9%)	54,310 (99.9%)	103 (100.0%)	
Acute Renal Failure				0.78
Yes	43 (0.1%)	43 (0.1%)	0 (0.0%)	
No	54,448 (99.9%)	54,345 (99.9%)	103 (100.0%)	
Urinary Tract Infection				0.30
Yes	517 (0.9%)	515 (0.9%)	2 (1.9%)	
No	53,974 (99.1%)	53,873 (99.1%)	101 (98.1%)	
CVA/Stroke				0.79
Yes	39 (0.1%)	39 (0.1%)	0 (0.0%)	
No	54,452 (99.9%)	54,349 (99.9%)	103 (100.0%)	
Cardiac Arrest				0.79
Yes	42 (0.1%)	42 (0.1%)	0 (0.0%)	
No	54,449 (99.9%)	54,346 (99.9%)	103 (100.0%)	
Myocardial Infarction				0.66
Yes	101 (0.2%)	101 (0.2%)	0 (0.0%)	
No	54,390 (99.8%)	54,287 (99.8%)	103 (100.0%)	
Bleeding Transfusion				0.001
Yes	2,201 (4.0%)	2,190 (4.0%)	11 (10.7%)	
No	52,290 (96.0%)	52,198 (96.0%)	92 (89.3%)	
Deep Venous Thrombosis				0.26
Yes	180 (0.3%)	179 (0.3%)	1 (1.0%)	
No	54,311 (99.7%)	54,209 (99.7%)	102 (99.0%)	
Sepsis				0.54
Yes	203 (0.4%)	203 (0.4%)	0 (0.0%)	
No	54,288 (99.6%)	54,185 (99.6%)	103 (100.0%)	
Septic Shock				<0.001
Yes	41 (0.1%)	41 (0.1%)	0 (0.0%)	
No	54,450 (99.9%)	54,347 (99.9%)	103 (100.0%)	
Any Medical Complication				0.01
Yes	4,525 (8.3%)	4,509 (8.3%)	16 (15.5%)	
No	49,966 (91.7%)	49,879 (91.7%)	87 (84.5%)

**Table 6:** Multivariable analysis of the impact of preoperative weight loss on postoperative outcomes in patients with BMI > 35

**Outcome**	**Odds Ratio/Coefficient**	**95% CI**	***P*-value**
Length of Stay (Days from Operation to Discharge)	β = 0.40	0.005–0.80	**0.047**
Bleeding Transfusion	OR = 2.60	1.37–4.93	**0.003**
Any Medical Complication	OR = 1.85	1.07–3.18	**0.03**

Bold values indicate *P* < 0.05

Patients who lost significant weight preoperatively had significantly increased LOS (β 0.40, 95% CI 0.005–0.80, *P* = 0.047), and were more likely to require a transfusion for bleeding (OR 2.60, 95% CI 1.37–4.93, *P* = 0.003) and experience any medical complication (OR 1.85, 95% CI 1.07–3.18, *P* = 0.03).

## Discussion

Numerous studies have shown that obesity is associated with adverse outcomes after THA, including increased LOS, readmission and higher revision rates, and higher rates of medical and surgical complications [3–5]. Consequently, many surgeons utilize BMI cutoffs to determine eligibility for THA [7, 8]. There has been a great deal of debate surrounding the benefits of preoperative bariatric surgery before THA, with studies showing mixed results in terms of impact on postoperative outcomes [10, 11]. The results of this study added to the limited literature regarding preoperative weight loss in obese patients, demonstrating that even when controlling for comorbidities, rapid substantial preoperative weight loss is not associated with improved outcomes and may, in some cases, be associated with worse outcomes. Furthermore, this study contributes novel evidence that preoperative weight loss in the general population is also associated with worse postoperative outcomes.

### General population

In the general population, significant preoperative weight loss was associated with an increased incidence of numerous adverse outcomes, including prolonged LOS, non-home discharge, readmission and reoperation, and medical complications. Notably, patients with preoperative weight loss were not predominantly obese at baseline, as reflected by a non-obese average BMI, and thus this increased incidence of adverse outcomes is not reflective of an increased risk for complications due to obesity. Although our analysis controlled for these factors, there were significant differences in comorbidities between patients with and without preoperative weight loss, which may have had an impact on the association with worse outcomes observed. For example, as patients with preoperative weight loss were significantly more likely to have low albumin levels suggestive of malnutrition, these effects may reflect the impact of malnutrition on outcomes. These results are in line with existing literature delineating the effects of malnutrition on various body systems. For example, malnutrition has been shown to adversely impact the immune system, leading to increased risk for infection and decreased ability to fight infection [15, 16], reduce kidney function [17], thus increasing risk of renal insufficiency, and increase risk of anemia [18], which may explain the increased incidence of bleeding complications. Increased intraoperative bleeding and musculoskeletal impacts of malnutrition may also increase complexity of procedures in patients with significant weight loss, thus increasing operative time. Finally, all the aforementioned adverse effects of malnutrition may have contributed to the increased rates of readmission and reoperation seen in this study.

That rapid substantial preoperative weight loss was associated with worse postoperative outcomes in the general population has important implications for clinical practice. As numerous preoperative demographics and comorbidities differed between the weight loss and non-weight loss groups, it follows that weight loss may be merely a proxy for underlying conditions that may predispose patients to poor postoperative outcomes. Orthopaedic surgeons should thus take note of significant preoperative weight loss in patients, especially when other signs of malnutrition are present, when determining patient eligibility for THA. Future research should be conducted to determine if malnutrition should be considered a relative contraindication for THA, and if preoperative nutritional restoration can mitigate postoperative complications after significant weight loss.

### Obese population

Rather than minimizing adverse postoperative outcomes, significant preoperative weight loss within obese patients was associated with an increased incidence of any medical complications, bleeding complications, and prolonged LOS. As this subgroup analysis contained only patients who were obese preoperatively, the baseline outcomes in both the weight loss and control cohorts should be reflective of the increased incidence of complications relative to non-obese patients noted in the literature [3–5]. Additionally, unlike for the general population, the majority of comorbidities did not significantly differ between patients who did and did not lose weight preoperatively, except only functional status, history of CHF, and renal failure. Consequently, any significant differences may reflect specifically the impact of weight loss on these outcomes. Obese patients who lost significant weight preoperatively were at increased risk of bleeding complications, which may reflect the aforementioned hematological impact of malnutrition. Notably, obese patients in the weight loss group were more likely to have preoperative albumin levels suggestive of malnutrition, providing further evidence of a potential impact of malnutrition on outcomes. The increased risk of medical complications may also have contributed to the prolonged LOS noted in obese patients who lost weight. An increased complication rate was also noted in a study by Inacio et al. that showed an increased rate of deep surgical site infection in obese patients who lost >5% of body weight compared to obese patients who maintained their weight [13]. This complication may not have been seen in our study given the smaller number of patients with the higher weight loss cutoff of >10% of body weight, or because our study controlled for comorbidities that may have predisposed patients to infection such as age and preoperative wound infections.

The findings of this study are of critical importance, as obesity has been highlighted as a risk factor for adverse outcomes, which may lead to the seemingly logical assumption that preoperative weight loss would improve outcomes. Consequently, arthroplasty surgeons may promote preoperative bariatric surgery or non-surgical weight loss as part of preoperative patient optimization. Study results have been mixed with respect to the impact of bariatric surgery on outcomes of THA, with some showing no difference and others showing an improvement in outcomes relative to other obese patients [10, 11]. However, the results of this study call into question the soundness of a recommendation for rapid preoperative weight loss. This question echoes that raised by Kim et al. in a study evaluating total knee arthroplasty patients, where preoperative weight loss was associated with rebound weight gain and increased risk for all-cause revision [19]. Consequently, orthopaedic surgeons must balance risks of rapid preoperative weight loss with risks of adverse outcomes in obese patients undergoing THA. In particular, the results raise the question of whether benefits of weight loss may be offset by the impact of malnutrition in the setting of rapid weight loss, and thus more work is needed to differentiate outcomes between those obese patients who lost weight who were and were not experiencing the effects of malnutrition. Future work should also determine if more gradual weight loss, which would likely not lead to patient malnutrition and be more sustainable, leads to fewer adverse outcomes than rapid weight loss.

### Strengths & limitations

This study represents a novel addition to the literature in terms of investigation of preoperative weight loss in the general population as well as investigation of this topic in obese patients while controlling for comorbidities and comparing to patients who remained obese as the control group. Strengths of this study include the use of a large, nationally representative database, which led to the ability to identify a wide array of demographics, comorbidities, and outcomes, patient BMI directly from their electronic medical records, and the implications the results have for clinical practice. However, although this study represents a valuable addition to the literature, we recognize that limitations exist. First, this study was a retrospective analysis and thus can only demonstrate association, not causation. Second, although NSQIP is a large, nationally representative database, the data are dependent on human coders and thus errors may exist. In addition, although weight information was collected directly from patient charts and should thus be relatively reliable, NSQIP does not provide data on the timing of preoperative weight collection, so it cannot be determined if weight data are accurate at the time of surgery. Similarly, only 0.2% of patients lost > 10% of body weight in the six months prior to surgery, which was very low, particularly among the obese patient population who may have been counseled on weight loss. This may reflect the difficulty patients face in achieving or sustaining significant weight loss prior to surgery, or that patients lost weight more gradually. However, it is also possible that some patients’ weight data six months prior to surgery were not available, and thus 0.2% may be an underestimate. Our analysis was also limited to variables available in the NSQIP database, and thus there may be factors, such as cause of weight loss (i.e., bariatric surgery vs. non-surgical weight loss), that contribute to incidence of adverse outcomes for which we were unable to control. However, as cause of weight loss is not able to be determined, it is important to note that weight loss may thus be considered a proxy for underlying cause. In particular, among non-obese patients, weight loss may indicate underlying comorbidities and malnutrition. Consequently, although this study cannot conclusively state that weight loss is the cause of poor outcomes, the results showed an association that highlights the importance of the consideration of weight loss as a proxy variable for any number of conditions that may worsen outcomes after THA. Similarly, with respect to obese patients, although the cause cannot be determined, the results indicated that weight loss was not associated with improved outcomes. Thus, in the setting of surgeon recommendations for preoperative weight loss, future work should determine if outcomes of THA after surgical weight loss differ from those after non-surgical weight loss in order to determine if either independently impacts outcomes. Additionally, NSQIP only includes information on 30-day outcomes, and thus differences in longer-term complications, readmissions, and revision surgeries could not be explored. Finally, the extremely small (0.2%) cohort of patients with significant preoperative weight loss may also have resulted in a lack of power for certain analyses. For example, numerous rare medical complications were not seen in the obesity subgroup analysis, so determination of the relative likelihood of these medical complications may only be possible in a larger sample size. Future work should investigate preoperative weight loss in a larger, matched cohort in order to be sufficiently powered to detect a greater degree of differences in outcomes between patients who do and do not lose weight preoperatively.

## Conclusions

In summary, the results of this study suggest that significant rapid preoperative weight loss is associated with adverse outcomes after THA. Although obese patients have been shown to have worse outcomes after joint arthroplasty, this study suggests that promotion of significant rapid preoperative weight loss is not associated with a reduction in the poor outcomes faced by this population and may in fact be associated with worse outcomes in some cases. The practice implications of these results are thus twofold: (1) orthopaedic surgeons should be aware of preoperative weight loss in patients and consider that significant rapid preoperative weight loss may be a relative contraindication to THA, and (2) when counseling obese patients, orthopedic surgeons need to balance risks of rapid preoperative weight loss with risks of performing THA in this patient population.

## Data Availability

The anonymised data collected in the NSQIP database are publicly available by request for download online as a Participant Use Data File at: https://www.facs.org/quality-programs/data-and-registries/acs-nsqip/participant-use-data-file/.
